# Development of a neural network-based automated eyelid measurement system

**DOI:** 10.1038/s41598-024-51838-6

**Published:** 2024-01-12

**Authors:** Yoonsoo Nam, Taekyung Song, Jaesung Lee, Jeong Kyu Lee

**Affiliations:** 1 Department of Ophthalmology, Chung-Ang University College of Medicine, Chung-Ang University Hospital, 102 Heukseok-ro, Dongjak-gu, Seoul, 06973 Korea; 2 Department of Artificial Intelligence, Chung-Ang University, Seoul, Korea

**Keywords:** Medical research, Diagnostic markers, Eye manifestations

## Abstract

The purpose of this study was to assess the clinical utility and reliability of an automated eyelid measurement system utilizing neural network (NN) technology. Digital images of the eyelids were taken from a total of 300 subjects, comprising 100 patients with Graves’ orbitopathy (GO), 100 patients with ptosis, and 100 controls. An automated measurement system based on NNs was developed to measure margin–reflex distance 1 and 2 (MRD1 and MRD2), as well as the lengths of the upper and lower eyelids. The results were then compared with values measured using the manual technique. Automated measurements of MRD1, MRD2, upper eyelid length, and lower eyelid length yielded values of 3.2 ± 1.7 mm, 6.0 ± 1.4 mm, 32.9 ± 6.1 mm, and 29.0 ± 5.6 mm, respectively, showing a high level of agreement with manual measurements. To evaluate the morphometry of curved eyelids, the distance from the midpoint of the intercanthal line to the eyelid margin was measured. The minimum number of divisions for detecting eyelid abnormalities was determined to be 24 partitions (15-degree intervals). In conclusion, an automated NN-based measurement system could provide a straightforward and precise method for measuring MRD1 and MRD2, as well as detecting morphological abnormalities in the eyelids.

## Introduction

Eyelid abnormalities can occur in various ophthalmic disorders, including Graves’ orbitopathy (GO), ptosis, and orbital tumors. For patients with eyelid abnormalities, it is necessary to evaluate the morphology and position of the eyelids. Various metrics, such as margin–reflex distance 1 (MRD1), MRD2, palpebral fissure height (PFH), and eyelid length, are currently being used to objectively assess the shape and condition of the eyelids^1,2^. MRD1 and MRD2 are conventional methods used to measure the vertical length of the eyelids. Upper and lower eyelid lengths are measured with the aid of digital images and software, which provide comprehensive information about the size and curvature of the eyelids. However, manually measuring parameters of the eyelid morphology has the drawback of being less consistent and reproducible. In addition, most of these metrics only capture the linear aspect of the eyelid contour, making it difficult to directly assess the morphology of curved eyelids. The concept of the mid-pupil lid distance (MPLD) has been used in various studies to compare the curvature of the eyelid between different patients^3–6^. However, when measuring the MPLD, it is necessary to establish a standardized horizontal line passing through the pupil. This requirement can sometimes lead to inconsistent outcomes.

In order to overcome the disadvantages of manual eyelid measurements, there have been attempts to automatically or semi-automatically evaluate eyelid morphology through digital image analysis^7–9^. However, semi-automatic measurements, similar to manual measurements, may introduce variability in the segmentation process. Automated measurements have the advantage of allowing consistent measurements through automated segmentation. However, if there is a low-contrast transition in the image, errors may occur during the edge detection process. Recently, neural networks (NNs) have been utilized in various fields of ophthalmology for image analysis^10,11^. NNs are not as limited by image contrast, and this limitation can be overcome by accurately detecting edges through the learning process. Additionally, they can also significantly reduce the time required for evaluation. Deep learning techniques have been reported as feasible in eyelid image analysis for the automated measurement of eyelid parameters^12–15^. If these NN techniques are designed to automatically and accurately establish the reference line, they will be able to provide more consistent and accurate measurements of the curved eyelid contour. In addition, the use of NNs to analyze eyelid images may be an effective approach for detecting outliers or abnormal areas, which provide immediate information on eyelid abnormalities.

In this study, we aimed to evaluate the clinical usefulness and reliability of a NN-based automated eyelid measurement system. We determined whether the contour of the eyelids could be measured using the intercanthal distance, which connects both canthi and is automatically established by the NNs as a reference line instead of the midpupillary line. Furthermore, we investigated whether the NN could effectively provide comprehensive information for detecting eyelid abnormalities during automated measurements.

## Results

The average age of the 300 subjects was 50.5 ± 18.6 years, and 204 (68.0%) of them were female. In subgroup comparison, the average age of the ptosis group was the highest at 63.9 ± 13.3 years, whereas the average age of the GO group was the lowest at 37.6 ± 12.1 years. The GO group had a significantly higher proportion of females compared with that in the normal group (*p* < 0.001) and the ptosis group (*p* = 0.047). However, there was no significant difference between the normal and ptosis groups (*p* = 0.596) (Table 1).

**Table 1:** Characteristics of subjects.

Variables	Total	Normal	Ptosis	Graves’ orbitopathy	*p*-value
	(N = 300)	(N = 100)	(N = 100)	(N = 100)	
Age (years)	50.5 ± 18.6	50 ± 19.3	63.9 ± 13.3	37.6 ± 12.1	< 0.001*
Sex					< 0.001**
Male, n (%)	96 (32.0%)	44 (44.0%)	34 (34.0%)	18 (18.0%)	
Female, n (%)	204 (68.0%)	56 (56.0%)	66 (66.0%)	82 (82.0%)	
MRD1 (mm)^#^	3.0 ± 1.5	3.1 ± 0.8	1.4 ± 0.6	4.4 ± 1.3	< 0.001*
MRD2 (mm)^#^	5.8 ± 1.3	5.8 ± 1.1	5.1 ± 1.2	6.4 ± 1.2	< 0.001*

MRD1, margin–reflex distance 1; MRD2, margin–reflex distance 2.

*ANOVA, pairwise t-test.

**chi-square test.

^#^measured by ImageJ.

We compared the values for 300 individuals obtained from both manual measurement and the proposed automated measurement system. The average values of MRD1, MRD2, upper eyelid length, and lower eyelid length were 3.2 ± 1.7 mm, 6.0 ± 1.4 mm, 32.9 ± 6.1 mm, and 29.0 ± 5.6 mm, respectively, when measured using the automated system and 3.0 ± 1.5 mm, 5.8 ± 1.3 mm, 31.5 ± 4.2 mm, and 28.4 ± 3.4 mm, respectively when measured manually. The average values obtained from the automated system were consistently higher than those obtained from manual measurement (Table 2).

**Table 2:** Comparison of neural network-based and manual measurements of eyelids.

Variables	Neural network (N = 300)	Manual (N = 300)	*p*-value
MRD1	3.2 ± 1.7	3.0 ± 1.5	< 0.001*
MRD2	6.0 ± 1.4	5.8 ± 1.3	< 0.001**
Upper eyelid length	32.9 ± 6.1	31.5 ± 4.2	< 0.001*
Lower eyelid length	29.0 ± 5.6	28.4 ± 3.4	0.005**

MRD1, margin–reflex distance 1; MRD2, margin–reflex distance 2.

* Wilcoxon rank-sum test.

**Paired t-test.

**Figure 1:**
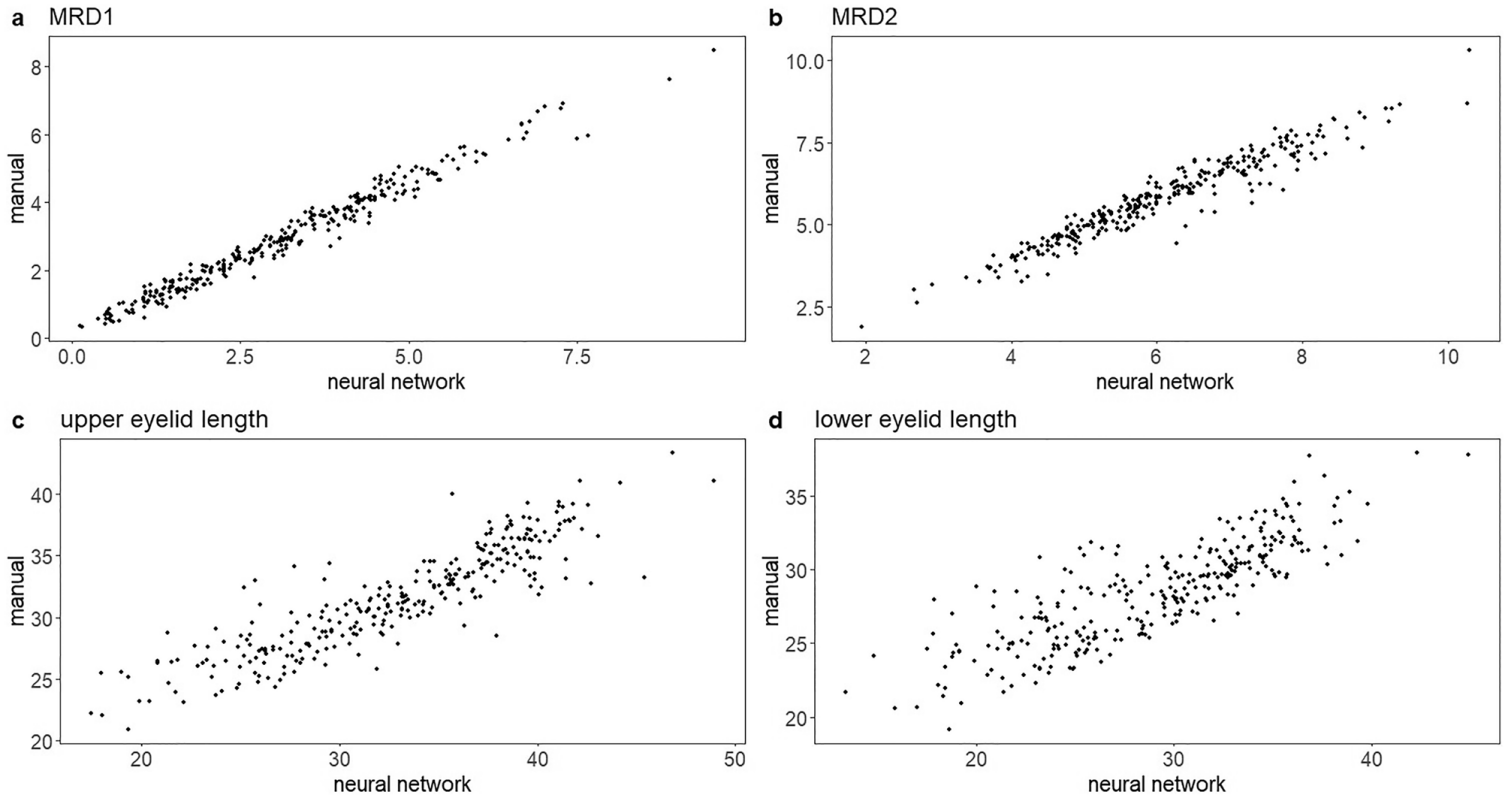
Correlation between the proposed automated measurement system and manual technique. (**a**) MRD1, (**b**) MRD2, (**c**) upper eyelid length (**d**) lower eyelid length.

The values obtained from both manual and automated measurements generally showed good agreement for all eyelid parameters of the subjects. The intraclass correlation coefficient (ICC) values indicated excellent correlation, with values of 0.972 for MRD1 and 0.937 for MRD2. However, the lengths of the upper and lower eyelids showed slightly lower values at 0.803 and 0.737, respectively (Fig. 1).

**Figure 2:**
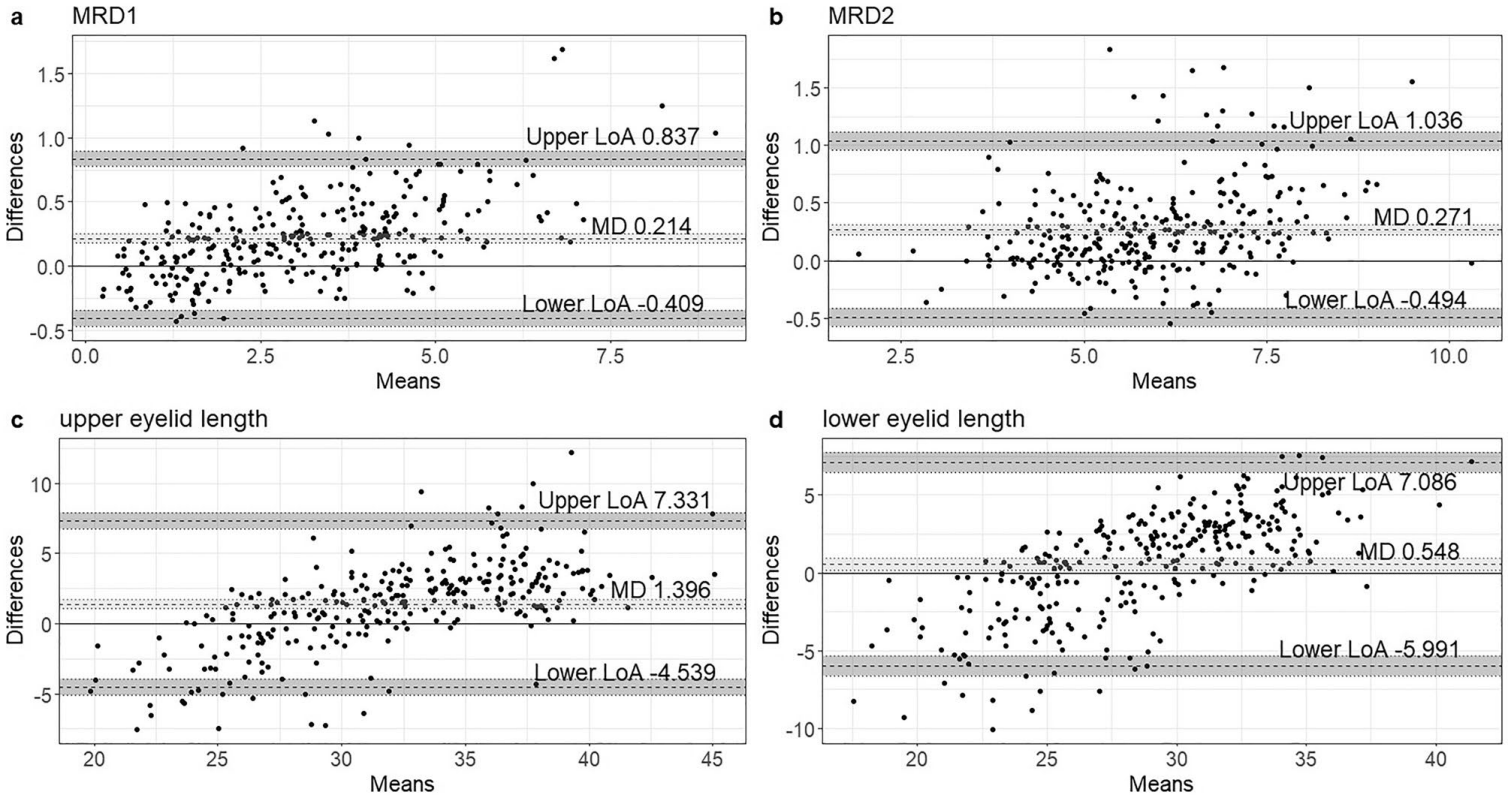
Bland Altman plots comparing the proposed automated measurement system and manual technique. MD, mean difference; LOA, limit of agreement.

The average differences and 95% limits of agreement for the values of all subjects obtained from manual and automated measurements were as follows: average difference of MRD1 = 0.214 mm (range: 0.178–0.250), average difference of MRD2 = 0.271 mm (range: 0.227–0.315), average difference of upper eyelid length = 1.396 mm (range: 1.052–1.740), and average difference of lower eyelid length = 0.548 mm (range: 0.169–0.927) (Fig. 2). The 95% limits of agreement for these measurements were narrow, indicating a high level of agreement between the values obtained from manual and automated measurements. All measured parameters exhibited statistically significant and strong correlations.

In subgroup analysis, both MRD1 and MRD2 consistently showed excellent agreement with ICC values of 0.9 or higher. On the other hand, the lengths of the upper and lower eyelids showed good agreement with ICC values of 0.747 and 0.772 in the normal control group and fair agreement with ICC values ranging from 0.553 to 0.650 in the ptosis and GO groups. The mean differences for MRD1 and MRD2 were 0.223 mm (range: 0.173–0.272) and 0.211 mm (range: 0.126–0.295), respectively, in the normal control group, 0.032 mm (range: − 0.018–0.082) and 0.251 mm (range: 0.173–0.330), respectively, in the ptosis group, and 0.388 mm (range: 0.320–0.455) and 0.351 mm (range: 0.284–0.417), respectively, in the GO group. These values were all less than 0.5 mm. The mean differences for the upper and lower eyelid lengths ranged from − 0.407 to 2.825 mm. The largest differences were observed in the GO group, with values of 2.633 mm and 2.825 mm, respectively (Supplementary Tables S1–S3).

**Figure 3:**
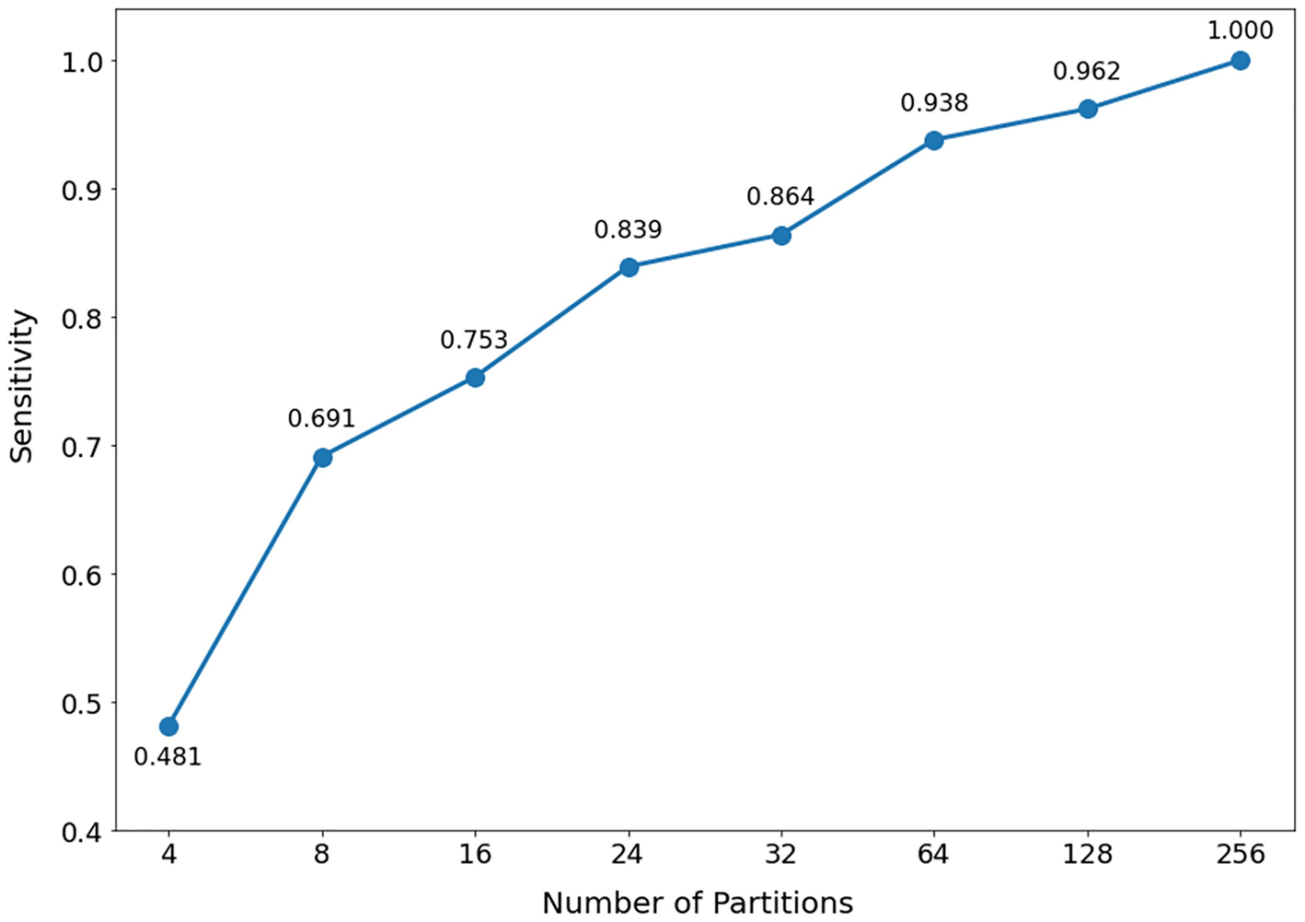
Experimental results evaluating the verification performance based on 4, 8, 16, 24, 32, 64, 128, and 256 partitions.

By evaluating the verification performance based on 4, 8, 16, 24, 32, 64, and 128 partitions, we obtained the ratio of identified abnormal subjects (Fig. 3). The ratio of identified abnormal subjects could be interpreted as the sensitivity of the test given that the number of identified abnormal subjects represents the number of true positives, whereas the total number of abnormal subjects is equal to the sum of the number of true positives and false negatives. The specificity of this test is always 100% because there are no false positive cases. Experimental results showed a sensitivity value above 0.8 from 24 partitions (15-degree intervals). However, the slope decreased from 32 partitions, indicating that the conventional 24 partitions could provide a good balance between test simplicity and verification performance.

## Discussion

In our study, we developed an automated eyelid measurement system based on NNs to quantitatively measure eyelid parameters, such as MRD1 and MRD2. We confirmed the accuracy of our system by comparing the data with those obtained from manual measurement. Current image measurement programs require a segmentation process, which can be either manual or semi-automated. However, this process often leads to problems such as interobserver variability, reduced reproducibility, and time consumption. We compared the results of a conventional image measurement method and an automated measurement method. The results showed a high degree of consistency between the two methods. Furthermore, the automated measurement method took only a few seconds and required no operator intervention.

Previous studies that performed deep learning-based image analysis of eyelid morphology have reported ICCs ranging from 0.934 to 0.971 for the MRDs of patients with ptosis^14^. For patients with GO, ICCs of 0.989 for MRD1 and 0.964 for MRD2 have been reported^12^. Our results also demonstrated that the proposed automated measurement system exhibited a high level of agreement with the manual measurement method for MRD1 and MRD2 in the overall group and individual subgroups (normal, ptosis, and GO), with ICC values exceeding 0.92. On the other hand, the agreement levels for both the length of the upper eyelid and the length of the lower eyelid were slightly lower (ranging from fair to good) compared with those for MRD1 or MRD2. This could possibly be attributed to the curvature of the eyelids. In our NN-based algorithm, pixels are linear; however, actual lengths have curves. Therefore, a slight error may have been introduced during the conversion process. In our study, we measure the lengths of both the upper and lower eyelids based on the pixel perimeter. However, using the pixel perimeter directly for length measurement may result in significant errors due to the square shape of a pixel; actual eyelids have a curved shape. Therefore, instead of using the pixel perimeter directly, we considered the quadrant perimeter to improve the accuracy of our measurements. This approach is important considering that relying solely on the pixel perimeter can result in significant differences in automated eyelid measurements compared with manual eyelid measurements. Moreover, an approach using three-dimensional reconstruction can improve the accuracy of length measurement by considering the curvature more precisely.

Based on the mean measurements, we found that our program yielded slightly high values for MRD1, MRD2, upper eyelid length, and lower eyelid length compared with those obtained from manual measurements. Although the exact reason for this discrepancy remains uncertain, it may be caused by differences in measurement strategies. The length based on the line connecting the center of the neighboring pixel was shorter than the length based on our approach using quadrants. Additionally, the agreement between the automated and manual measurements was higher in the normal group but lower in the ptosis or GO group. A possible explanation for this observation is the difficulty of performing accurate measurements for subjects with ptosis or GO. Patients with ptosis or GO have eyelid margins that are more uneven and curved than those of normal patients. These characteristics make it difficult to obtain accurate measurements.

In contrast to MPLD measurements performed in previous studies to assess the shape of curved eyelids^5,16,17^, we measured the distance from the midpoint of the intercanthal line to the eyelid margin. Our rationale for this method is twofold. First, when subjects do not maintain primary gaze, the pupil light reflex may not align with the center, potentially compromising the objectivity of the results. Second, when assessing the eyelid shape exclusively, measuring from the midpoint of the intercanthal line has important implications. As NNs can clearly define the two points on each canthus that form the intercanthal line, the intercanthal line would be more easily and objectively established as the reference line. This line is expected to yield more consistent values compared with those obtained using the horizontal midpupillary line. However, with this approach, the intercanthal line often does not form a straight line, with the lateral canthus typically positioned slightly higher, resulting in an inclined line. To address this, we rotated the inclined intercanthal line to make it aligned horizontally using simple mathematics. Furthermore, when using this approach for measurements, the distances from the pupil, MRD1, and MRD2, are not included.

The conventional approach of dividing the eyelids (360 degrees) into 15-degree intervals (24 partitions) has been used to characterize the entire eyelid contour; however, the exact rationale has not been provided^3^. In order to achieve precise evaluation, this study aimed to determine the most accurate eyelid segmentation approach for detecting abnormalities in the eyelids. The minimum number of divisions was determined to be 24 partitions, which is consistent with the traditional method using 15-degree intervals. This number may be considered the most appropriate for evaluating abnormal eyelid shapes in our study.

Our study has several limitations. First, the sample size consisted of only 300 subjects. Moreover, the selection process was not completely random. Ideally, it would be beneficial to include a wide range of diseases and a larger sample size of subjects. A random selection from a larger pool, which would reflect the overall distribution of diseases, could yield more informative results. Another limitation is that length measurement was based on the corneal light reflex due to camera flash, which is not applicable when the MRD1 value is negative. Additional research is needed to automatically and accurately estimate the location of the pupil center, which can serve as a reference instead of relying on the light reflex.

In conclusion, we proposed an automated NN-based measurement system that could provide a straightforward and precise method for measuring MRD1 and MRD2, as well as detecting morphological abnormalities in the eyelids. Similar to MPLD measurement, measuring the distance from the midpoint of the intercanthal line to the eyelid margin was also useful in evaluating the shape of curved eyelids. Furthermore, eyelid segmentation showed that the meaningful detection of abnormalities could be achieved with 24 partitions. This novel system may be used to provide comprehensive information to detect eyelid abnormalities by adopting it for other eyelid-related diseases during automated measurements. Our developed system could be implemented in portable devices such as cameras or smartphones for potential clinical use. Alternatively, it could be integrated into existing medical devices or used as a standalone application for wider accessibility. Further studies will be needed for clinical applications in the future.

## Methods

The protocol was approved by the Institutional Review Board of Chung-Ang University Hospital (IRB No. 2303-014-19461). The requirement for informed consent was waived due to the retrospective nature of the study. The study was conducted in accordance with the principles of the Declaration of Helsinki. Consent for publication has been obtained from the individual depicted in the illustration featured in this manuscript.

### Image collection

The study was conducted retrospectively using electronic medical records from January 2010 to February 2023, with a specific focus on outpatient ophthalmology visits at Chung-Ang University Hospital. A total of 100 patients with a normal eyelid morphology, 100 patients with eyelid retraction due to GO, and 100 patients with eyelid ptosis were included in the study. Normal controls were subjects who visited our clinic for reasons such as dry eye syndrome, cataract, or a regular check-up for retinal examination. Patients with unclear diagnoses or those whose pupillary reflex was not visible in facial photographs were excluded from the study. Patients who had undergone previous upper or lower eyelid surgery were also excluded. Demographic information, such as age and gender, was recorded.

The facial photographs were taken by a single examiner in the same room under identical conditions using a 12.3-megapixel automated digital camera (Nikon D90, Nikon, Tokyo, Japan). The aperture, shutter speed, and exposure time were determined based on external lighting conditions. Subjects were instructed to relax and focus on the center of the camera lens in the primary gaze position. A circular marker with a diameter of 9.0 mm was placed on the subjects’ forehead before the image was taken to provide a reference scale in millimeters per pixel. The images were then transferred to a personal computer and saved as JPG files (1200 × 797 pixels, 24-bit, RGB).

### Manual measurement of eyelid parameters

Quantitative measurements of eyelid parameters were performed with patient photographs using ImageJ software version 1.46 (National Institutes of Health, Bethesda, MD, USA; http://rsbweb.nih.gov/ij/). The following parameters were measured and analyzed: MRD1, which is the vertical distance between the upper eyelid margin and the corneal light reflex; MRD2, which is the vertical distance between the lower eyelid margin and the corneal light reflex; upper eyelid length, which is the curvilinear length of the upper eyelid between the medial and lateral canthus; lower eyelid length, which is the curvilinear length of the lower eyelid between the medial and lateral canthus. The measurements were performed by two of the authors (Y.S.N., and J.K.L.) independently. The average values of all parameters were used for data analysis. Among the eyelids of both eyes, the left eyelid or the eyelid with more severe symptoms was selected for comparison of automated and manual measurement.

### Automated measurement of eyelid parameters

**Figure 4:**
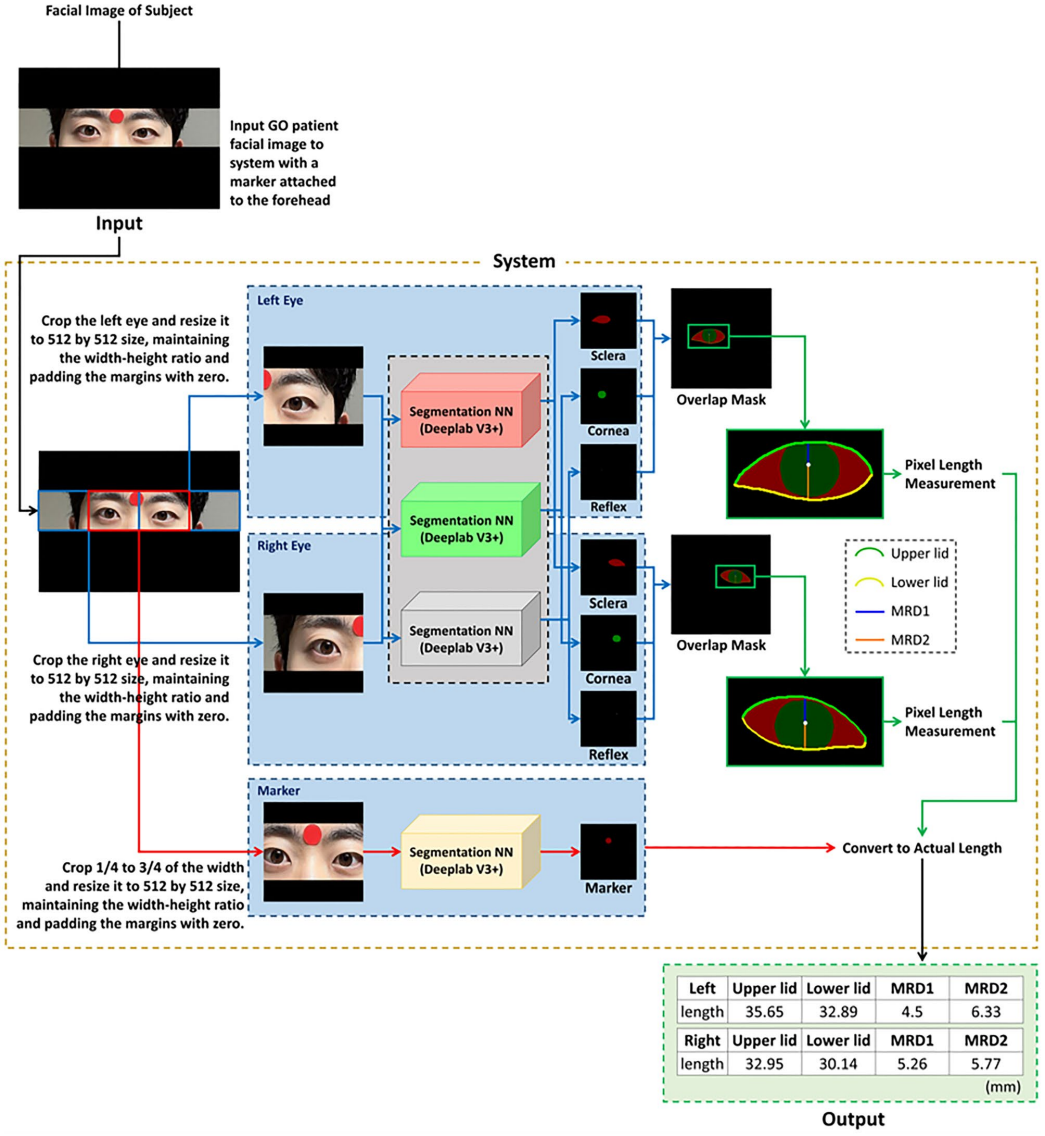
Eyelid segmentation.

An automated measurement system based on NNs was developed to measure MRD1 and MRD2, as well as the lengths of the upper and lower eyelids (Fig. 4). First, the system divided the original image of the subjects into three images, each showing the left eye, right eye, or circular marker. The left and right eye images were obtained by vertically splitting the original image in half. The marker image was obtained by cropping the area between 1/4 and 3/4 of the original image’s width. Next, the three images were resized to a square image of 512 × 512 pixels. To prevent distortion caused by resizing, the system maintained the original width-to-height ratio and filled any remaining areas with zero padding. Then, the left and right eye images were fed into three NN models (DeepLab V3+)^18^, with each model responsible for outputting the segments of the sclera, cornea, and light reflex. Therefore, they were individually trained to segment the sclera, cornea, and light reflex. One additional DeepLab V3 + model was used for marker segmentation. The diameter of the segmented marker was then used to convert the pixel length of the sclera, cornea, and light reflex into their respective actual lengths in millimeters (mm).

After image segmentation, each segment was overlaid by the system for the length measurement process. In the first step, the length of the outer boundary of the cornea was measured. The horizontal endpoints of the segmented sclera were used as reference points to differentiate between the upper and lower eyelids. In this study, we measured the length of the eyelids using the perimeter of a virtual quadrant instead of directly measuring the pixel perimeter. which was performed when the pixels on the eyelid formed a corner (Supplementary Fig. S1). Using a proportionality equation, the actual length of the eyelids could also be calculated. The midpoint of the light reflex layer was used to calculate the vertical distance between the upper and lower eyelids. The diameter of the marker was used to calculate MRD1 and MRD2 with proportional equations. The results were then compared with manually measured values.

### Automated detection of eyelid abnormalities

**Figure 5:**
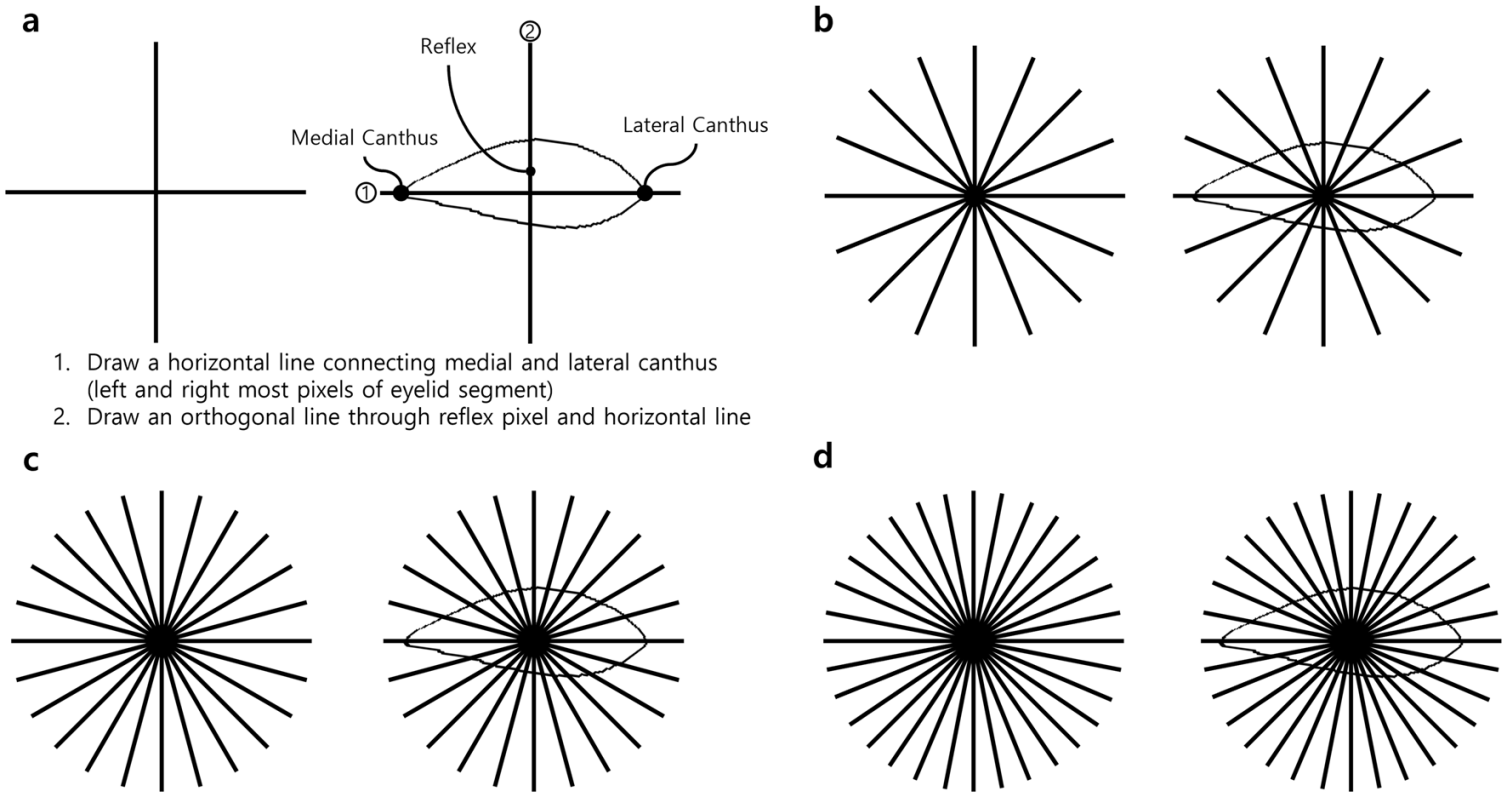
Eyelid segmentation. (**a**) 4 partitions (90.0-degree intervals), (**b**) 16 partitions (22.5-degree intervals), (**c**) 24 partitions (15.0-degree intervals), and (**d**) 32 partitions (11.3-degree intervals).

The system divided the eyelid margin into 360 degrees (1-degree intervals). First, a horizontal line was added by connecting pixels on the lateral and medial canthi. Then, an orthogonal line was drawn by connecting a pixel on the light reflex with the horizontal line (Fig. 5). As a result, we could obtain four partitions by drawing two lines. Additional partitions could be obtained by dividing each partition equally, based on the given degree intervals. Our system then measures the distance from the point where the intercanthal line intersects with the vertical line passing through the pupil light reflex to the eyelid margin for each segment. In this study, the corresponding degree segment was considered abnormal if the length was outside the 95% range of the normal distribution for 300 subjects.

Additionally, any subject who had at least one abnormal segment of 360 degrees was classified as an abnormal subject. The verification performance for abnormal subjects could be improved by reducing the degree interval. Therefore, an optimal trade-off between the degree interval and the verification performance could be achieved. In this study, we determined the ratio of identified abnormal subjects to true cases by varying the degree interval as follows: 90.0, 45.0, 22.5, 15.0, 11.3, 5.6, 2.8, and 1.4; for simplicity, we defined each degree interval as having 4, 8, 16, 24, 32, 64, 128, and 256 partitions, respectively.

To train DeepLab V3 + for image segmentation, 724 images were used. These images included the left and right eye, pupil reflex, and marker of the subjects. For the two eye images, we applied left–right augmentation to improve the accuracy of the learning process. These images were randomly divided into training, validation, and test sets with ratios of 0.6, 0.2, and 0.2, respectively. The hyperparameters were set as follows: a batch size of 32, 30 epochs, AdamW optimizer, and a learning rate of 1e−3. We assessed the performance of DeepLab V3 + using the mean Intersection over union (mIoU) metric. We conducted all the experiments using the PyTorch (version 1.10.1) library and a computer system equipped with a GeForce RTX 3090 24 GB GPU (NVIDIA, Santa Clara, CA, USA).

### Statistical analysis

Data are represented as the mean value with standard deviation. We performed analysis of variance (ANOVA) to analyze demographic characteristics such as age and gender, as well as quantitative measurements including MRD1, MRD2, upper eyelid length, and lower eyelid length. The difference between automated and manual measurements was assessed by Wilcoxon rank-sum test and paired t-test. The ICC was calculated to assess the agreement between manual and automated measurements. ICC values below 0.40 were categorized as 'poor', and values in the range of 0.40–0.60, 0.60–0.75, and 0.75–1.00 were characterized as 'fair', 'good', and 'excellent', respectively. Bland–Altman plots were used to visualize the discrepancies between manual and automated measurements. The 95% confidence intervals for the mean difference and limits of agreement (LOA) are shown on the plot. All statistical analyses were performed using R software (version 4.2.2). A *p*-value of < 0.05 was considered statistically significant.

### Supplementary Information

Supplementary Information.

## Data Availability

Datasets used and analyzed during the current study are available from the corresponding author upon reasonable request.
