# Supplementary material for: Development of a neural network-based automated eyelid measurement system
Source: Sci Rep. 2024 Jan 12;14:1202. doi: 10.1038/s41598-024-51838-6 (PMC10786902; doi:10.1038/s41598-024-51838-6)
Supplement: Supplementary file 1 — Supplementary Information. [file 41598_2024_51838_MOESM1_ESM.pdf]

Development of a Neural Network-based Automated Eyelid Measurement System

Yoonsoo Nam[1], Taekyung Song[2], Jaesung Lee[2*], Jeong Kyu Lee[1*]

[1]Department of Ophthalmology, Chung-Ang University College of Medicine, Chung-Ang University Hospital, Seoul, Korea

[2]Department of Artificial Intelligence, Chung-Ang University, Seoul, Korea

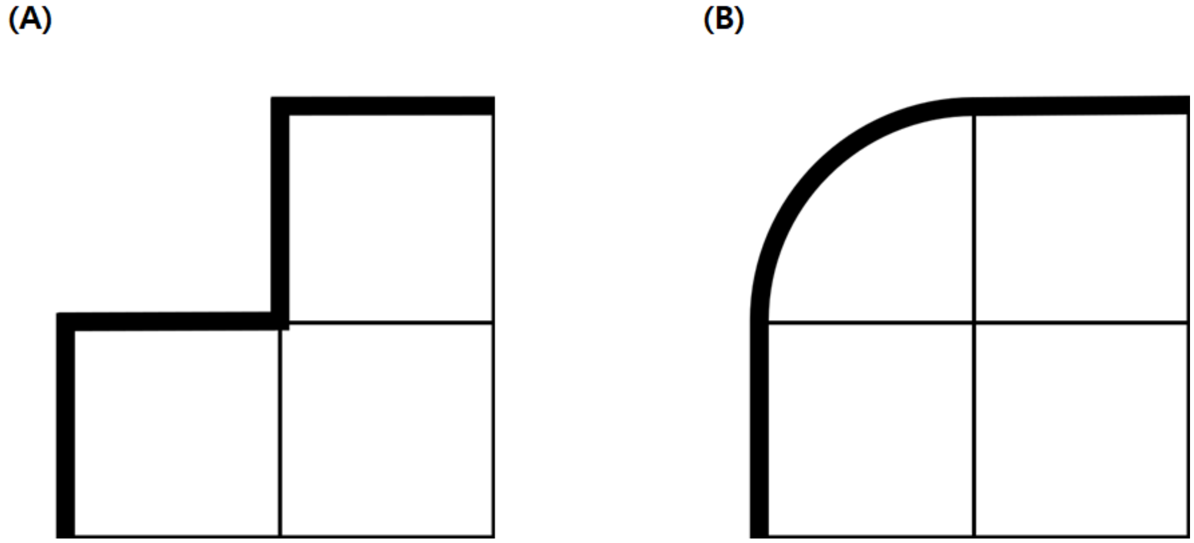

Figure S1. The difference between pixel perimeter (A) and virtual quadrant perimeter employed in our system (B). The length based on the pixel perimeter is 4 whereas the length of the virtual perimeter is approximately 3.57.

Table S1. Average differences and 96% limits of agreement of lid parameters in normal controls

	ICC	MD	Lower LoA	Upper LoA
MRD1	0.920 (0.634 to 0.969)	0.223 (0.173 to 0.272)	-0.265 (-0.349 to -0.180)	0.710 (0.173 to 0.272)
MRD2	0.911 (0.827 to 0.950)	0.211 (0.126 to 0.295)	-0.621 (-0.765 to -0.476)	1.042 (0.897 to 1.186)
Upper lid length	0.747 (0.386 to 0.876)	1.963 (1.446 to 2.480)	-3.141 (-4.027 to -2.255)	7.068 (6.182 to 7.954)
Lower lid length	0.772 (0.676 to 0.841)	0.583 (0.068 to 1.097)	-4.496 (-5.377 to -3.614)	5.661 (4.779 to 6.542)

MRD, margin-reflex distance; LoA, limits of agreement.

Table S2. Average differences and 96% limits of agreement of lid parameters in patients with ptosis

	ICC	MD	Lower LoA	Upper LoA
MRD1	0.920<br>(0.884 to 0.946)	0.032<br>(-0.018 to 0.082)	-0.465<br>(-0.551 to -0.379)	0.529<br>(0.442 to 0.615)
MRD2	0.930<br>(0.819 to 0.966)	0.251<br>(0.173 to 0.330)	-0.526<br>(-0.661 to -0.391)	1.029<br>(0.894 to 1.164)
Upper lid length	0.650<br>(0.521 to 0.750)	-0.407<br>(-1.120 to 0.306)	-7.449<br>(-8.672 to -6.227)	6.634<br>(5.412 to 7.857)
Lower lid length	0.553<br>(0.332 to 0.703)	-1.765<br>(-2.470 to -1.060)	-8.730<br>(-9.939 to -7.521)	5.2<br>(3.991 to 6.410)

MRD, margin-reflex distance; LoA, limits of agreement.

Table S3. Average differences and 96% limits of agreement of lid parameters in patients with Graves' orbitopathy

	ICC	MD	Lower LoA	Upper LoA
MRD1	0.930<br>(0.464 to 0.977)	0.388<br>(0.320 to 0.455)	-0.276<br>(-0.392 to -0.161)	1.052<br>(0.936 to 1.167)
MRD2	0.924<br>(0.526 to 0.974)	0.351<br>(0.284 to 0.417)	-0.307<br>(-0.422 to -0.193)	1.009<br>(0.895 to 1.123)
Upper lid length	0.650<br>(-0.075 to 0.876)	2.633<br>(2.298 to 2.967)	-0.674<br>(-1.248 to -0.100)	5.939<br>(5.365 to 6.513)
Lower lid length	0.602<br>(-0.080 to 0.849)	2.825<br>(2.447 to 3.203)	-0.907<br>(-1.555 to -0.259)	6.557<br>(5.909 to 7.205)

MRD, margin-reflex distance; LoA, limits of agreement.
